# Longitudinal changes in sedentary time and physical activity during adolescence

**DOI:** 10.1186/s12966-015-0204-6

**Published:** 2015-04-01

**Authors:** Sarah K Harding, Angie S Page, Catherine Falconer, Ashley R Cooper

**Affiliations:** Centre for Exercise, Nutrition and Health Sciences, University of Bristol, Bristol, UK; National Institute for Health Research, Bristol Biomedical Research Unit in Nutrition, Diet and Lifestyle, Bristol, UK

**Keywords:** Adolescents, Sedentary time, Light physical activity, Moderate to vigorous physical activity

## Abstract

**Background:**

Low levels of physical activity and high time spent in sedentary activities have been associated with unfavourable health outcomes in adolescents. During adolescence, physical activity declines and sedentary time increases, however little is known about whether the magnitude of these changes differs within or between school-time, after-school time, or at weekends.

**Methods:**

Adolescents (n = 363) participating in the PEACH (Personal and Environmental Associations with Children’s Health) project provided accelerometer data at 12 and 15 years of age. Data were collected in 2008/2009 and 2012/2013. Time spent sedentary (<100 cpm), in light physical activity (LPA (100-2295 cpm) and in moderate to vigorous physical activity (MVPA: ≥ 2296 cpm) were generated for school-time, after-school time and for weekends using school-specific start and finish times. All data were analysed in 2014.

**Results:**

The proportion of time spent sedentary significantly increased during school (+8.23%, 95% CI = 7.35 to 9.13), after-school (+6.99%, 95% CI = 5.91 to 8.07) and at weekends (+6.86%, 95% CI = 5.10 to 8.62). A parallel decrease was found in the proportion of time spent in LPA during school (-7.62%, 95% CI = -8.26 to -6.98), after-school (-7.01%, 95% CI = -7.74 to -6.28) and at weekends (-6.72%, 95% CI = -7.80 to -5.65). The proportion of time spent in MVPA remained relatively stable during school (-0.64, 95% CI = -1.11 to -0.18), after-school (0.04%, 95% CI = -0.58 to 0.67) and at weekends (-0.14%, 95% CI = -1.18 to 0.90).

**Conclusions:**

Objectively measured sedentary time increased between 12 and 15 years of age during-school, after-school, and at weekends, suggesting that interventions aiming to reduce the age-associated changes in sedentary time are needed in all three time contexts. Future work should identify which sedentary activities change more than others to inform interventions which aim to minimise the increase in time spent sedentary during adolescence.

## Background

Low physical activity (PA) in youth is associated with a range of adverse health outcomes [1,2] however, relatively few adolescents meet physical activity guidelines [3,4] In addition, there is emerging evidence that sedentary behaviours in adolescence are negatively associated with adolescent health outcomes such as obesity and metabolic risk [5-7] although the evidence for this using objective measures are inconsistent [8-10]. Sedentary time may also track from adolescence into adulthood [11], where it has consistently been associated with unfavourable health outcomes such as metabolic risk, cardiovascular disease and all-cause mortality [12-14] thus extending the public health benefits of reducing sedentary time in adolescents. Furthermore, greater sedentary time may reduce time available for moderate to vigorous intensity physical activity (MVPA) [6,9]. Physical activity declines by an estimated 7% per year through adolescence [15], and there is a concurrent increase in sedentary time (ST) [16-18] with some authors suggesting an increase of 90 minutes/day from early to late adolescence [17].

Identifying the correlates, determinants and different contexts (such as time) within which sedentary behaviours and physical activity are undertaken is an important stage in the development of interventions [19-21]. Although a range of studies have investigated the correlates of physical activity and sedentary time, temporal factors such as when sedentary time and physical activity occur have received little attention [19]. Nonetheless, the evidence which does exist suggests that there are important differences in the level of sedentary time and physical activity performed by adolescents both between days of the week (weekday vs weekend day) and within the weekday day (school-time vs non-school time). Brooke et al. [19] systematically reviewed and meta-analysed the literature on time specific differences in objectively measured physical activity in school children aged 4-18 years. They compared total physical activity and MVPA between time segments (weekday vs weekend day, in school vs out of school, weekend vs out of school and lessons vs break times). The results showed that children were more active (with regard to both total physical activity and MVPA) on weekdays compared with weekend days. There were also significant differences in both total physical activity and MVPA during the school day; total physical activity was lower in school compared with out of school whereas MVPA was higher in school compared with out of school. The authors suggest that this is because although schools have opportunities to engage in higher intensity physical activity such as during PE lessons, there is also the inherent requirement to be sedentary for a large portion of school time [19]. However, these data were not presented as a proportion of time and thus, due to the different time frames these are not directly comparable. In addition, it is worth noting that they did not investigate age differences and as the demands of school increase with age it is expected that these patterns would differ by age.

Longitudinal (age associated) changes in sedentary time and physical activity may differ according to the time of day, since when out of school, with the exception of active commuting, physical activity and sedentary behaviours are more likely to be discretionary compared with school time where opportunities to be physically active are limited to school breaks, lunch times and physical education lessons. However, few studies have investigated whether longitudinal changes in physical activity and sedentary time vary between different periods of the day. Arundell et al. [22] segmented accelerometer data to explore the 5-year changes in after-school sedentary time and physical activity in participants aged 10-12 years at baseline. In both males and females after-school sedentary time increased and light intensity physical activity (LPA), moderate intensity physical activity and vigorous intensity physical activity declined significantly. However, these authors did not explore changes in sedentary time and physical activity at other times, such as during-school or at weekends.

An understanding of the time context of the change in sedentary time and physical activity through adolescence could inform interventions to prevent the age associated changes in these. Therefore, the purpose of this paper is to describe the longitudinal changes in the level of objectively measured sedentary time, LPA and MVPA during school-time, after-school and at weekends over three years in adolescents aged 12 years at baseline, and to assess if the magnitude of change differs between the school, after-school and weekend segments.

## Methods

### Participants

Data were from the PEACH (Personal and Environmental Associations with Children’s Health) project in Bristol, UK. The study was approved by a University Ethics Committee and all participating children provided written informed consent from a parent/guardian. The PEACH project was longitudinal in design and consisted of three phases. Details of study design have been reported elsewhere [23]. The current study is focused on the last two phases, in which participants were aged 12 years (school year 7) and 15 years (school years 10 and 11). Participants were from 27 secondary schools in and around Bristol, UK. To control for seasonality, data were collected throughout the year but collected at the same time of year for each participant in 2008/2009 and 2012/2013.

### Measurement of sedentary time and physical activity

Sedentary time and physical activity were measured using accelerometers (GT1M; ActiGraph LLC, FL, USA) worn on a belt around the waist during waking hours for seven days. Height was measured using a stadiometer and weight was measured using digital scales (SECA). Body mass index (BMI) was calculated as (weight(kg)/height(m)^2^) and weight status categories (healthy weight, overweight, obese) were computed using UK age and sex-specific tables [24]. Index of multiple deprivation (IMD) scores were determined from participants’ home postcodes. IMD is an measure of deprivation that includes income, health, education and employment status of the geographical area around home postcodes [25], tertiles of these socres were created within the dataset.

Accelerometer data were processed using Kinesoft (v3.3.62; KineSoft, Saskatchewan, Canada). Continuous periods of ≥60 minutes of zero values, allowing for 2 minutes of interruption, were considered to be non-wear time and were discarded. Time spent sedentary (sedentary time), in LPA and in MVPA were computed using established thresholds (sedentary time: <100 cpm, LPA: 100-2295 cpm, MVPA: ≥ 2296 cpm) [26,27]. Weekday accelerometer data were segmented into during-school and after-school segments (ending at 9 pm), based upon the specific start and end times of each school (n = 27). For inclusion in analyses, participants were required to provide ≥60% of the total possible time available during-school (average possible time across schools was 397.7 minutes) and after-school (average possible time across school was 339.3 minutes), on at least one full day at both time points. A ≥60% wear-time criteria was chosen for each time segment as this is approximately equivalent to the widely used validated 600 minute/day wear time criteria [28] (which is 62.5% of a 16 hour waking day). Participants in the weekend analysis wore the accelerometer for ≥480 minutes on at least one day, in addition to meeting the weekday wear-time criteria.

### Statistical analysis

Values for sedentary time, LPA and MVPA were standardised to 100% of the possible wear-time for school and after-school (specific for each school) segments e.g. minutes of MVPA accumulated during school-time were divided by actual minutes of wear during school-time and then multiplied by possible minutes of wear during school-time. This was to control for differences in wear time both between segments and between phases. Weekend data were standardised to the mean weekend wear-time (698 minutes). Change in time (minutes) spent in sedentary, in LPA and in MVPA were calculated for each time point. The proportion of time spent in each physical activity intensity was then computed for each segment to allow for the differences in time frames between segments.

Paired samples T-tests were used to examine longitudinal differences in the proportion of time spent in sedentary time, LPA and MVPA in each time segment between baseline and follow-up. Owing to the possibility of clustering within schools (n = 27), random effects mixed models were used to determine if the proportion (%) of time spent in sedentary time, LPA and MVPA changed differentially between school-time, after-school and weekends. Data were checked for normal distribution visually using histograms. Continuous data are presented as means ± SD. Data were analysed in 2014 using STATA/IC (version 12.1).

## Results

Nine hundred and fifty three participants took part in phase 2 of the PEACH project. Of these 485 (50.89%) also took part in phase 3. Three hundred and sixty three (74.85%) participants met the accelerometer weekday wear time criteria at both time points and were included in the analysis. See Table 1 for the descriptive characteristics of the participants. At both time points there was no significant difference in the age of those who met the weekday wear time criteria compared with those who did not. However, those who met the weekday accelerometer wear time criteria had a lower BMI, IMD and were more likely to be female, at both time points (p < 0.05 for all comparisons), compared to those who did not meet the wear time criteria. Of those that met the weekday wear time criteria, 199 participants (41.03%) also met the weekend wear-time criteria at both time points and were included in the weekend analysis. Those who met the weekend wear time criteria had a lower BMI, IMD and were more likely to be female, at both time points (p < 0.05 for all comparisons) when compared with those who did not meet the weekend wear time criteria. There was no evidence that change in time (both proportion of time and minutes) spent sedentary, in LPA and in MVPA differed significantly between males and females for during school-time, after-school or weekends (p > 0.05 for all comparisons). Thus, data from males and females were combined. The mean follow-up time was 3.36 years (referred to as 3 years herein).

**Table 1:** **Descriptive characteristics of the study sample at ages 12 and 15 years (n = 363)**

		**12 years**		**15 years**	
		**Females**	**Males**	**Females**	**Males**
Number of participants		223	140	223	140
Age (mean (SD))		12.0 (0.4)	12.0 (0.4)	15.4 (0.5)	15.3 (0.5)
BMI (mean (SD))		19.6 (3.8) Range 13.3 to 37.2	18.4 (2.9) Range 14.0 to 29.5	21.5 (5.5) Range 15.9 to 39.8	20.6 (3.5) Range 15.1 to 34.6
BMI	Healthy weight (n (%))	171 (76.7)	119 (85)	159 (76.8)	111 (84.7)
	Overweight (n (%))	41 (18.4)	16 (11.4)	34 (16.4)	14 (10.7)
	Obese (n (%))	11 (5.0)	5 (4.0)	14 (6.8)	6 (4.6)
IMD score	Lower (least deprived) (n (%))	113 (60.1)	76.35 (65.0)	113 (60.1)	76.35 (65.0)
	Middle (n (%))	57 (30.3)	35 (29.9)	57 (30.3)	35 (29.9)
	Upper (most deprived) (n (%))	18 (9.6)	6 (5.13)	18 (9.6)	6 (5.1)

BMI: body mass index, IMD: index of multiple deprivation.

Longitudinal changes in sedentary time, LPA and MVPA are presented in Table 2. There was a significant increase in the proportion of time spent sedentary during school (+8.23%, 95% CI = 7.35 to 9.13), after-school (+6.99%, 95% CI = 5.91 to 8.07) and at weekends (+6.86%, 95% CI = 5.10 to 8.62). There were significant decreases in the proportion of time spent in LPA during-school (-7.62%, 95% CI = -8.26 to -6.98), after-school (-7.01%, 95% CI = -7.74 to -6.28) and at weekends (-6.72%, 95% CI = -7.80 to -5.65). With regard to MVPA, there was a significant change in the proportion of time spent in MVPA during school (-0.64, 95% CI = -1.11 to -0.18) this equates to a decrease of 2.5 minutes/day. There was no significant change in the proportion of time spent in MVPA after-school (0.04%, 95% CI = -0.58 to 0.67) or at weekends (-0.14%, 95% CI = -1.18 to 0.90).

**Table 2:** **Changes in sedentary time, LPA and MVPA (proportion of time and minutes)**

			**12 years Mean(SD)**	**15 years Mean(SD)**	**Difference (95% CI) Mean(SD)**	**P Value of difference** ^**a**^
Proportion of time (%)	Sedentary time (%)	School	66.22 (8.06)	74.46 (7.87)	+8.23 (7.35 to 9.13)	<0.001
		After-school	63.15 (9.75)	70.14 (9.31)	+6.99 (5.91 to 8.07)	<0.001
		Weekends^b^	64.45 (9.00)	71.43 (10.37)	+6.86 (5.10 to 8.62)	<0.001
	Light PA (%)	School	27.21 (6.26)	19.58 (5.87)	-7.62 (-8.26 to -6.98)	<0.001
		After-school	27.82 (6.59)	20.80 (6.10)	-7.01 (-7.74 to -6.28)	<0.001
		Weekends^b^	28.24 (6.10)	21.53 (6.67)	-6.72 (-7.80 to -5.65)	<0.001
	MVPA (%)	School	6.60 (3.80)	5.95 (3.53)	-0.64 (-1.11 to -0.18)	0.007
		After-school	9.00 (4.95)	9.04 (5.59)	+0.04 (-0.58 to 0.67)	0.893
		Weekends^b^	7.31 (4.32)	7.03 (6.35)	-0.14 (-1.18 to 0.90)	0.582
Time (minutes) in each intensity	Sedentary time (minutes)	School	263.9 (36.2)	295.9 (35.7)	+32.0 (28.4 to 35.6)	<0.001
		After-school	213.7 (34.1)	238.5 (33.7)	+24.8 (21.1 to 28.5)	<0.001
		Weekends^b^	449.6 (62.8)	498.4 (72.4)	+48.8 (36.9 to 60.6)	<0.001
	LPA (minutes)	School	108.1 (24.4)	77.7 (23.1)	-30.4 (-32.9 to -27.9)	<0.001
		After-school	94.5 (23.8)	71.0 (22.0)	-23.5 (-26.0 to -21.0)	<0.001
		Weekends^b^	197.5 (42.2)	150.6 (46.7)	-46.9 (-54.4 to -39.4)	<0.001
	MVPA (minutes)	School	26.3 (14.9)	23.8 (14.4)	-2.5 (-4.4 to -0.7)	0.007
		After-school	30.6 (17.2)	31.0 (19.7)	+0.4 (-1.8 to 2.5)	0.724
		Weekends^b^	50.7 (30.1)	49.8 (45.1)	-1.0 (-8.2 to 6.3)	0.582

LPA: light intensity physical activity, MVPA: moderate to vigorous intensity physical activity. ^a^P value is for paired samples t-tests testing difference between age 12 years and 15 years. ^b^for those in the weekend sample n = 199.

The results of the random effects mixed models showed that there was no evidence that the magnitude of change in sedentary time differed between school-time and after-school (B = -1.25, 95% CI = -2.728 to 0.234), between school and weekend (B = -1.097, 95% CI = -2.821 to 0.627) and between after-school and weekend (B = 0.149, 95% CI = -1.573 to 1.874). There was also no evidence of a difference in the magnitude of change in LPA between school and after-school, between school and weekend or between after-school and weekend (B = 0.612, 95% CI = -0.381 to 1.605, B = 0.864, 95% CI = -0.291 to 2.02, and B = 0.252, 95% CI = -0.903 to 1.407 respectively). Finally no evidence was obtained that suggested that the magnitude of change in MVPA differed between school and after-school, between school and weekend and between after-school and weekend (B = 0.688, 95% CI = -0.155 to 1.531, B = 0.279, 95% CI = -0.702 to 1.260, B = -0.408, 95% CI = -1.390 to 0.573, respectively).

## Discussion

This paper describes the 3-year changes in sedentary time and physical activity from age 12 years in a sample of UK adolescents. The results provide evidence to suggest that some school-time, after-school and weekend LPA is displaced by sedentary time from ages 12 to 15 years. The results suggest that there is no difference in the magnitude of change between school-time, after-school and weekends, thus interventions to reduce the age associated changes in sedentary time are needed in all three segments.

To the author’s knowledge, this is the first longitudinal study to segment accelerometer data into school-time, after-school and weekend segments to describe when any changes in sedentary time, LPA and MVPA may occur. Cross-sectional differences in physical activity between segments of the day (school verses out of school) and of the week (weekday verses weekend) have previously been described [19]. The current study extends this by identifying when in the day and week sedentary time increases and physical activity (specifically LPA) decreases during adolescence.

This study found only small changes in MVPA over the 3 year follow-up. Although the changes in MVPA presented here may not be representative of the changes in adolescent the population more widely due to sample selection, these findings are similar to a recent study using a similar population which found that, over a 4 year follow-up (from ages 12 years to 16 years), the absolute levels of MVPA remained relatively stable in both males and females [17]. The findings of a pooled analysis [15] which consisted of 26 longitudinal studies showed that physical activity decreases by 7% per year during adolescence. However, this review did not look specifically at MVPA and the majority of the papers included (21 out of 26) used self-reported measures of physical activity and only 2 used accelerometers. Together these may suggest that MVPA remains relatively stable during adolescence and the changes seen in the review reflect changes in light-intensity physical activity which would be consistent with the findings of the current study and of Mitchell et al. [17].

The result of this study showed that on weekdays sedentary time increases by approximately an hour and by approximately 50 minutes on weekend days, and that similar inverse changes were seen in LPA. This increase in sedentary time during adolescence agrees with the results of Mitchell at al. [17] which found that sedentary time increases from the ages of 12 years to 16 years, and also those of Treuth et al. [29] who found that sedentary time increased from 462 minutes/day to 510 minutes/day in adolescent girls from age 12 to 14 years and that there was a parallel decrease in LPA. Several studies have supported the notion that specific sedentary behaviours such as TV viewing and computer use are associated with poor health in adolescents [7,30,31]. However, when accelerometers have been used to measure total time spent sedentary the evidence is less consistent; a review of longitudinal studies concluded insufficient evidence of an association between the volume of sedentary behaviour and metabolic risk factors in children and adolescents [10]. Despite this, in adults, there is consistent evidence that time spent sedentary is associated with poor cardiometabolic risk factors [12-14]. Since there is evidence which shows that levels of sedentary behaviour in adolescence tracks moderately well into adulthood [11] the growing consensus that sedentary time increases during adolescence is of concern.

### Strengths and limitations

The study has a number of strengths. School-time and after-school segments were created using the specific start and finish times for each school, allowing for accurate representation of the adolescent’s day. Previous cross-sectional papers, which have segmented physical activity data, have used generic school start and end times [32-34], which may introduce substantial misclassification. For example, Gidlow et al. [35] compared in-school and out of school activity patterns generated using school start and finish times with “school related” and “after-school” physical activity using a fixed cut point of time before/after 4 pm. Physical activity was significantly higher during the “school related” segment, since this included both after-school extra-circular activities and active travel to/from school.

In addition, for inclusion in analyses it was required that participants wore the accelerometer for at least 60% of the possible wear-time for school and after-school for at least one full day to ensure that each segment was accurately represented. This approach overcomes the limitation of applying a fixed minimum period of accelerometer wear-time for inclusion, for example 480 minutes [23,36,37] which may not capture and represent all segments of the day equally. In a study of adults, participants were found to be more likely to wear accelerometers during the day and remove them in the evening [38]. Thus, when a daily wear time criteria is applied evening sedentary time and physical activity would be under-represented. This may also be true for adolescents as they may be more likely to wear the accelerometers during school-time and remove them in the evenings, resulting in an over-representation of school time. Applying segment specific wear-time criteria ensures that sedentary time and physical activity during all the segments of interest are represented.

There are also limitations to this study. A consequence of the stringent wear-time criteria is a reduced sample size, and potentially the overrepresentation of some groups compared to others; participants were more likely to meet the wear-time criteria if they were female, had lower BMI or were of higher socioeconomic position. Thus, the results of this study may suffer from inclusion bias, and may not be more widely generalizable. In addition, all participants were recruited from schools in Bristol. Therefore results may not be able to be generalized to other cities.

## Conclusions

This study shows that sedentary time increases and LPA decreases during adolescence. It extends existing literature by showing when the changes occur. It was found that changes in sedentary time and LPA activity occur both during weekdays (during-school and after-school) and during the weekends. This suggests that interventions during-school, after-school and at weekends have the potential to minimise the age associated changes in sedentary time and LPA. It also suggests that, since the changes occur both during school and out of school, there are changes in a range of activities that may contribute to the increase in sedentary time. Further studies are required to describe which activities change more than others in order to inform interventions to reduce sedentary time, and hence increase physical activity.

## Abbreviations

MVPA: Moderate to vigorous physical activity
LPA: Light intensity physical activity
BMI: Body mass index
IMD: Index of multiple deprivation
